# Supplementary material for: Mother-daughter asymmetry of pH underlies aging and rejuvenation in yeast
Source: eLife. 2014 Sep 4;3:e03504. doi: 10.7554/eLife.03504 (PMC4175738; doi:10.7554/eLife.03504)
Supplement: Supplementary file 1. — (A) Yeast Strains. (B) Oligonucleotides. DOI: http://dx.doi.org/10.7554/eLife.03504.010 [file elife03504s001.docx]

**Supplementary file 1A: Yeast Strains**

| **Strain** | **Genotype** |
| --- | --- |
| UCC8773 | MATa his3Δ1 leu2Δ0 ura3Δ0 lys2Δ0 hoΔ::P_SCW11_-cre-EBD78-NatMX loxP-CDC20-Intron-loxP-HphMX loxP-UBC9-loxp-LEU2 |
| UCC8774 | MATα his3Δ1 leu2Δ0 ura3Δ0 trp1Δ63 hoΔ::P_SCW11_-cre-EBD78-NatMX loxP-CDC20-Intron-loxP-HphMX loxP-UBC9-loxp-LEU2 |
| UCC4925 | MATa/MATα his3Δ1/his3Δ1 leu2Δ0/leu2Δ0 ura3Δ0/ura3Δ0 lys2Δ0/+ trp1Δ63/+ hoΔ::P_SCW11_-cre-EBD78-NatMX/hoΔ::P_SCW11_-cre-EBD78-NatMX loxP-CDC20-Intron-loxP-HphMX/loxP-CDC20-Intron-loxP-HphMX loxP-UBC9-loxp-LEU2/loxP-UBC9-loxp-LEU2  (Diploid created from UCC8773 and UCC8774 parental haploid strains) |
| UCC4963 | UCC4925 VPH1-mCherry-KanMX/+ |
| UCC5480 | MATa/MATα his3Δ1/his3Δ1 leu2Δ0/leu2Δ0 ura3Δ0/ura3Δ0 hoΔ::P_SCW11_-cre-EBD78-NatMX/hoΔ::P_SCW11_-cre-EBD78-NatMX loxP-CDC20-Intron-loxP-HphMX/loxP-CDC20-Intron-loxP-HphMX loxP-UBC9-loxp-LEU2/loxP-UBC9-loxp-LEU2 VMA2-GFP-HIS3MX6/VMA2-GFP-HIS3MX6 |
| UCC9645 | UCC4925 PMA1-mCherry-KanMX/+ |
| UCC9646 | UCC4925 chrI(17068-17161)P_ADH1_-PSR1-pHluorin-URA3/chrI(17068-17161)P_ADH1_-PSR1-pHluorin-URA3 |
| UCC9647 | UCC4925 CDC10-mCherry-KanMX/+ |
| UCC9648 | MATa/MATα his3Δ1/his3Δ1 lys2∆0/+ met15∆0/+ ura3∆0/ura3∆0 leu2∆0:: P_ACT1_-GAL4dbd-ER-VP16ad-NatMX/leu2∆0:: P_ACT1_-GAL4dbd-ER-VP16ad-NatMX VPH1-mCherry-KanMX/+ |
| UCC9649 | UCC9648 chrI(199456-199457)::P_GAL1_-PMA1-Term_CYC1_-URA3/chrI(199456-199457)::P_GAL1_-PMA1-Term_CYC1_-URA3 |
| UCC9650 | MATa/MATα his3Δ1/his3Δ1 lys2∆0/+ met15∆0/+ ura3∆0/ura3∆0 leu2∆0:: P_ACT1_-GAL4dbd-ER-VP16ad-NatMX/leu2∆0:: P_ACT1_-GAL4dbd-ER-VP16ad-NatMX Pma1-mCherry-KanMX/+ |
| UCC9651 | UCC9650 chrI(199456-199457)::P_GAL1_-PMA1-mCherry-Term_CYC1_-URA3/chrI(199456-199457)::P_GAL1_-PMA1-mCherry-Term_CYC1_-URA3 |
| UCC9652 | UCC4925 URA3/URA3 VPH1-mCherry-KanMX/+ |
| UCC9653 | UCC4925 pma1-105-URA3/pma1-105-URA3 VPH1-mCherry-KanMX/+ |
| UCC9654 | MATa/MAT@ his3Δ1/his3Δ1 leu2Δ0/leu2Δ0 lys2Δ0/lys2Δ0 hoΔ::P_SCW11_-cre-EBD78-NatMX/hoΔ::P_SCW11_-cre-EBD78-NatMX loxP-CDC20-Intron-loxP-HphMX/loxP-CDC20-Intron-loxP-HphMX loxP-UBC9-loxp-LEU2/loxP-UBC9-loxp-LEU2 |
| UCC9655 | MATa/MAT@ his3Δ1/his3Δ1 leu2Δ0/leu2Δ0 ura3Δ0/ura3Δ0 lys2Δ0/lys2Δ0 hoΔ::P_SCW11_-cre-EBD78-NatMX/hoΔ::P_SCW11_-cre-EBD78-NatMX loxP-CDC20-Intron-loxP-HphMX/loxP-CDC20-Intron-loxP-HphMX loxP-UBC9-loxp-LEU2/loxP-UBC9-loxp-LEU2 pma1-105-URA3/pma1-105-URA3 |
|  |  |
| UCC9656  UCC9657 | UCC8773 chrVII(479252-479253)URA3  MATa/MAT@ his3Δ1/his3Δ1 ura3Δ0/ura3Δ0 met15∆0/+ leu2∆0:: P_ACT1_-GAL4dbd-ER-VP16ad-NatMX/leu2∆0:: P_ACT1_-GAL4dbd-ER-VP16ad-NatMX chrI(17068-17161)P_ADH1_-PSR1-pHluorin-URA3/chrI(17068-17161)P_ADH1_-PSR1-pHluorin-URA3 |
| UCC9658 | UCC9657 chrI(199456-199457)::P_GAL1_-PMA1-Term_CYC1_-URA3/chrI(199456-199457)::P_GAL1_-PMA1-Term_CYC1_-URA3 |
| UCC9659 | MATa/MAT@ his3Δ1/his3Δ1 leu2Δ0/leu2Δ0 lys2Δ0/+ chrI(17068-17161)P_ADH1_-PSR1-pHluorin-URA3/chrI(17068-17161)P_ADH1_-PSR1-pHluorin-URA3 |
| UCC9660 | MATa/MAT@ his3Δ1/his3Δ1 leu2Δ0/leu2Δ0 ura3∆0/ura3∆0 lys2Δ0/+ chrI(17068-17161)P_ADH1_-PSR1-pHluorin-URA3/chrI(17068-17161)P_ADH1_-PSR1-pHluorin-URA3 pma1-105-URA3/pma1-105-URA3 |
| UCC9663 | UCC9654 vma2::kanMX/vma2::kanMX |
| UCC9664 | UCC9655 vma2::kanMX/vma2::kanMX |
| UCC9665 | UCC4925 Pma1-GFP-KanMX/+ |
| UCC10436 | UCC4925 Vph1-GFP-KanMX/Vph1-GFP-KanMX |
|  |  |

**Supplementary file 1B: Oligonucleotides**

| **Name** | **Sequence** |
| --- | --- |
| ChrI PartA NotI F | GTCCCATTCGAAGAAGCGGCCGCTTTAGCTCATTGAGATATGTG |
| ChrI PartB SmaI F | CGTCAATGCAAGCCCGGGCCATGGATGGTCGTTTAAGGC |
| ChrI PartB NotI R | TCTCAATGAGCTAAAGCGGCCGCTTCTTCGAATGGGACCAGCTA |
| ChrI PartA SmaI R | GTATTCTACGACCCCGGGGGTGCTAATTATGGCATTGAT |
| GibsonRXNpAGdstnF-2 | ctgtacaagtagTATACACAGCCAGTCTGCAG |
| GibsonRXNpAGdstnR-2 | gaggatgatgtatcagtcatGATCCACTAGTTCTAGAATC |
| GibRXNpAGdest_Pma1ChryF | gattctagaactagtggatcATGACTGATACATCATCCTC |
| GibRXNpAGdest_Pma1ChryR | Ctgcagactggctgtgtatactacttgtacagctcgtccatgc |
| MRKdownPma1F | TCGTCTTTATTATGGTCAAGGCTTTACGTCATAATAGTTCGATTGTACTGAGAGTGCACC |
| MRKdownPma1R | CGAGAAGAGATCTTTAAAGTATTATAGAAGAGCTGGGCAGCTGTGCGGTATTTCACACCG |
| Pma1_1kbupNotIF | gcggtggcggccgcCTCAGCTTTGCTAAAGTGCAAAAAGTCGTTTACG |
| Pma1_1kbdownSacIIR | gatcccgcggCCAGGGTAGTATACTATAGAAGGTCAGACTAAAC |
| Pma1F5 | GACTTCATGGCTGCTATGCAAAGAGTCTCTACTCAACACGAAAAGGAAACCggtgacggtgctggttta |
| Pma1R3 | GATTAAAATGTGACAAAAATTATGATTAAATGCTACTTCAACAGGATTAtcgatgaattcgagctcg |
| pma1-S368FF | gttcaaaagttgtctgctattgaattcttggctggtgtcg |
| pma1-S368FR | Cgacaccagccaagaattcaatagcagacaacttttgaac |
| PSR1-28-RMpHluorinF | Ctgacggtgctggtttaattaacatgggtttcatatcgtcaatactgtgctgctcttccgagacgacacaatccaattccaattctgcttatcgccaacaacagagcatgagtaaaggagaagaactttt |
| PSR1-28-RMpHluorinR | aaaagttcttctcctttactcatgctctgttgttggcgataagcagaattggaattggattgtgtcgtctcggaagagcagcacagtattgacgatatgaaacccatgttaattaaaccagcaccgtcag |
| SEP PacI F | CTGGTTTAATTAACATGAGTAAAGGAGAAGAACTTTTCA CTG |
| SEP AscI R | GAAGTGGCGCGCCTTATTTGTATAGTTCATCCATGCC |
| UPGFP/phluorin F | gatcgaattctgacggtgctggtttaattaac |
| UPGFP/phluorin R | gatccggccggacgaggcaagctaaacagatc |
| URA3-tTA-intChr1F | GTACGTCTACAGAGTATAGTGTAGAAATTATAGTTAGGATTGCATCAGAGCAGATTGTAC |
| URA3-tTA-intChr1R | GTATAAGTATCAATACCAATCACCATCTGACCATAAACTCTCGAGGTATTGGATAGTTCC |
| VMA2 D5 | AGAGTAGACAGTACATCAAGCGAAAATAAATATTGCAGGAGATTGTACTGAGAGTGCACC |
| VMA2 D3 | AAAATAAAAAAAGCCTTTTTCTTCAGCAACCGTCCTCTTACTGTGCGGTATTTCACACCG |
|  |  |
|  |  |
|  |  |
|  |  |
|  |  |
|  |  |
